# Bloodwork-free Early Screening for Alzheimer’s Disease via Comorbid Pattern Recognition in Electronic Health Records

**DOI:** 10.21203/rs.3.rs-8789582/v1

**Published:** 2026-02-09

**Authors:** Dmytro Onishchenko, James A. Mastrianni, Ishanu Chattopadhyay

**Affiliations:** 1 Division of Biomedical Informatics, Department of Internal Medicine, University of Kentucky, Lexington, KY, USA; 2 Department of Neurology, University of Chicago, Chicago, IL USA; 3 Director, Memory Center, University of Chicago, Chicago, IL USA

## Abstract

Early identification of Alzheimer’s disease and related dementias (ADRD) remains limited by the need for specialized tests and late-stage diagnosis. The Zero-burden Risk Assessment (ZeBRA) is a AI-driven score that predicts incident ADRD up to a decade before diagnosis, using only routine electronic health record (EHR) data, without laboratory tests, imaging, or questionnaires. Trained on 487,989 cases and 12,483,718 controls from nationwide U.S. insurance claims and validated on held-back samples, and two independent cohorts, ZeBRA achieved AUC = 0.93 and 0.83 for predicting out to 1-year and 10-year horizons respectively, maintaining positive likelihood ratios (>10) at 95% specificity and stable discrimination over time (AUC drop ≈ 1 to 1.3% per year). Performance was consistent across age, sex, race, and ethnicity subgroups. In a limited prospective pilot, higher ZeBRA scores correlated with lower Montreal Cognitive Assessment (MoCA) scores, indicating a greater degree of cognitive impairment (R=-0.78). Compared with prior EHR-based models, ZeBRA provides superior accuracy, cross-site generalizability, and demonstrates noise-corrected interpretability via our novel Λ-OR attrubution metric. Its scalability, low cost, and independence from specialized testing position ZeBRA as a practical tool for population-level early detection and presymptomatic trial enrichment.

## Introduction

Alzheimer’s disease (AD), the leading cause of dementia, accounts for 60–80% of cases worldwide^1,2^. The global burden of AD and related dementias (ADRD) has more than doubled since 1990, with prevalence projected to reach 13.8 million in the United States by 2050 and over 80 million worldwide by 2040^3–6^. In the U.S. alone, ADRD contributed to over 120,000 deaths in 2022 and more than $400 billion in annual unpaid caregiving costs^4^. Despite decades of research, therapeutic progress has been modest, with recent agents such as lecanemab and donanemab showing only limited benefits^4^.

AD is now suspected to have a prolonged presymptomatic phase during which molecular pathology accumulates in the absence of overt cognitive symptoms^7,8^. The 2024 NIA-AA framework redefined AD biologically, based on amyloid or tau positivity regardless of clinical presentation^9,10^. However, biomarker-based diagnostics are still expensive and/or invasive, and particularly impractical for population-wide screening, making direct adoption of this criteria difficult in practice. Blood-based assays show promise but remain unapproved for routine use^4^. These limitations underscore the need for passive, scalable, and cost-effective tools that can identify at-risk individuals before cognitive decline^11–14^.

Here we present the Zero-burden Risk Assessment (ZeBRA) algorithm, that analyzes routinely collected electronic health records (EHRs) to detect longitudinal comorbidity patterns predictive of a future ADRD diagnosis, realizing a reliable non-invasive point-of-care screening tool. Unlike existing approaches, ZeBRA requires no laboratory testing, imaging, or questionnaires, enabling low-cost, near-instantaneous risk assessment. Using multiple large, heterogeneous datasets, we demonstrate that ZeBRA predicts ADRD onset with clinically useful accuracy up to ten years in advance, and is robust across age, sex, race, and baseline risk. These findings support its potential as a presymptomatic screening tool for population health management and for enriching clinical trial cohorts with high-risk individuals.

ZeBRA models the joint comorbidity structure of diagnosis, prescription, and procedure codes in patient EHRs, achieving a 5–8 % absolute AUC gain over the nearest competitor^15^, sustained over a decade-long horizon across four independent data sources. The model remains stable across demographically disparate populations and provides robust, literature-consistent risk attribution through a noise-corrected odds-ratio metric (the Λ-OR) that enables an interpretable robust universal point-of-care screening tool for early diagnosis of ADRD.

## Results

### Datasets:

We evaluated ZeBRA on four cohorts: (1) the Merative MarketScan^®^, 2007–2024^36^) comprising over seven billion time-stamped diagnosis codes and tracking over 96 million patients for 1–16 years, referred to as the National dataset; (2) a de-identified patient cohort from the University of Chicago Medical Center, comprising patients treated between 2006 – 2021 (Chicago); (3) the NIH *All of Us* Research Program, comprising patient data collected between 2000 – 2023 (All Of Us), with > 80% participants historically underrepresented in biomedical research^37^; and (4) data from a limited prospective pilot at UChicago (UCM Pilot).

We formulated the prediction of a future diagnosis as a binary classification problem of patients’ time-stamped sequences of diagnostic codes into case and control subcohorts. We defined cases as individuals who met at least one of the following criteria: (1) a recorded diagnosis of Alzheimer’s disease and related dementias (ADRD), identified via corresponding International Classification of Diseases, Tenth Revision (ICD-10) diagnostic codes (see Supplementary Table SI-I, we focus on ADRD codes exclusively following literature^15^, and do not target mild cognitive impairment); or (2) a prescription record for an anti-dementia medication (see Supplementary Table SI-II). Control patients were defined as individuals with no diagnoses or prescriptions specified in the case definition, and with no recorded conditions known to be associated with or causally related to dementia (See Supplementary Table SI-III).

**Figure 1.**
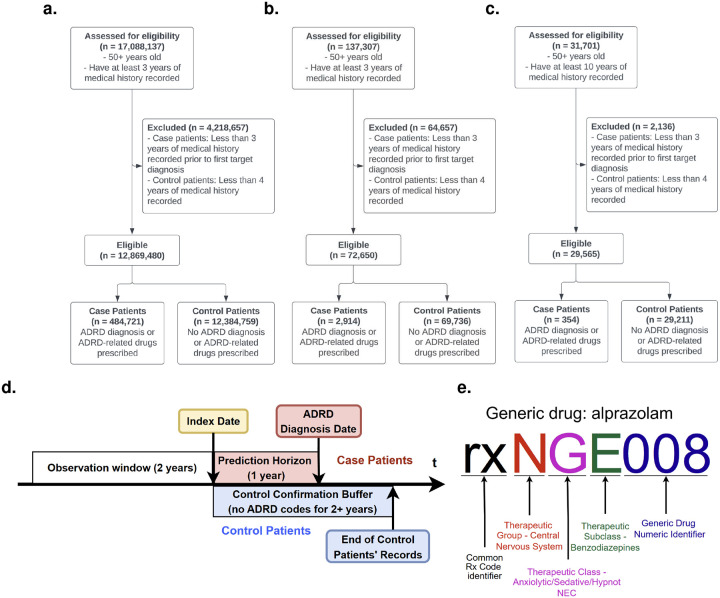
CONSORT diagrams and prediction timeline. a-c, shows inclusion and exclusion decisions for the three retrospective data sources. d, illustrates the timeline of observation, index and 1 year prediction horizon settings for the main results. e, Example of RX code for the generic drug alprazolam using Micromedex RED BOOK^©^

For National, Chicago, and All Of Us we included patients aged ≥ 50 years with ≥ 3 years of continuous history; we excluded cases with < 3 years before first ADRD code and controls with < 4 years of history. To address All Of Us heterogeneity, we required ≥ 10 years of records. CONSORT diagrams for National/Chicago/All Of Us and UCM Pilot appear in Figures 1 and 3a. In total, n=12,971,707 with $n_{\text{case}}=487,989$. We used 66% of National for training and 34% for primary out-of-sample validation; Chicago, All Of Us, and UCM Pilot were reserved entirely for independent validation (Table I).

For case patients the index date was 1 year before the first ADRD code, yielding a 1-year *Prediction Window*. For controls, the index date was 2 years before end-of-record, yielding a 2-year *control confirmation buffer* to mitigate misclassification. For both, the model training period or the *Observation Window* was the 2 years preceding the index date (Fig. 1d).

### Model and Evaluation:

We used three EHR modalities within each observation window: diagnoses (DX), generic prescriptions (RX), and procedures (PROC) without pre-selection, leveraging all available code sets and abbreviations. ZeBRA comprises six LightGBM base models^38,39^ (presence and odds-ratio embeddings for DX, RX, PROC) whose outputs, together with sex and age, feed a final classifier producing the ADRD risk score (training/validation workflows in Supplementary Figures SI-I, SI-II). Metrics include AUC, sensitivity, specificity, PPV, NPV, accuracy, and likelihood ratios (LR^+^, LR^–^); LR^+^ ≥ 10 indicates strong diagnostic utility^40–42^. High-risk and low-risk strata are defined by presence/absence of known ADRD comorbidities^16,18,21–25,28,29,32,34^ (Supplementary Table SI-IV). To harmonize prevalence across datasets, performance is reported assuming a 10.9% ADRD prevalence (1 in 9)^4^. While the true prevalence in the broader 50+ population is lower, positive and negative predictive values scale approximately linearly with prevalence, whereas discrimination metrics (AUC, sensitivity, specificity, likelihood ratios) are prevalence-invariant; we therefore adopt a fixed 10.9% prevalence for all datasets to enable direct performance comparison, consistent with prior EHR-based studies including Li *etal*.^15^, which report results under a similar prevalence assumption.

### Predictive Performance:

We measure our performance using standard metrics including the Area Under the receiver-operating characteristic curve (AUC), sensitivity, specificity, Positive Predictive Value (PPV), Negative Predictive Value (NPV), and accuracy. We also report positive likelihood ratio (LR+) that indicates how much a positive screening result increases the odds of a patient to be diagnosed with ADRD in future, and negative likelihood ratio (LR-) that indicates how much a negative screening result decreases those odds. While lower limit of a clinically meaningful positive likelihood ratio is 2,^40^ positive likelihood ratios of 10 and higher are generally indicative of a screening tool with strong diagnostic utility and high clinical significance.^41,42^

To demonstrate performance stratified for different ADRD risk groups, we define the high-risk subcohort as patients having one or more conditions in the observation window that are known ADRD co-morbidities^16,18,21–25,28,29,32,34^ (See Supplementary Table SI-IV), and a low-risk subcohort as patients that have none of these conditions recorded in the observation window. Results in the low risk sub-cohort are of particular significance, because these patients lack common risk factors of ADRD and are at a higher risk of delayed or missed diagnosis.

**Figure 2.**
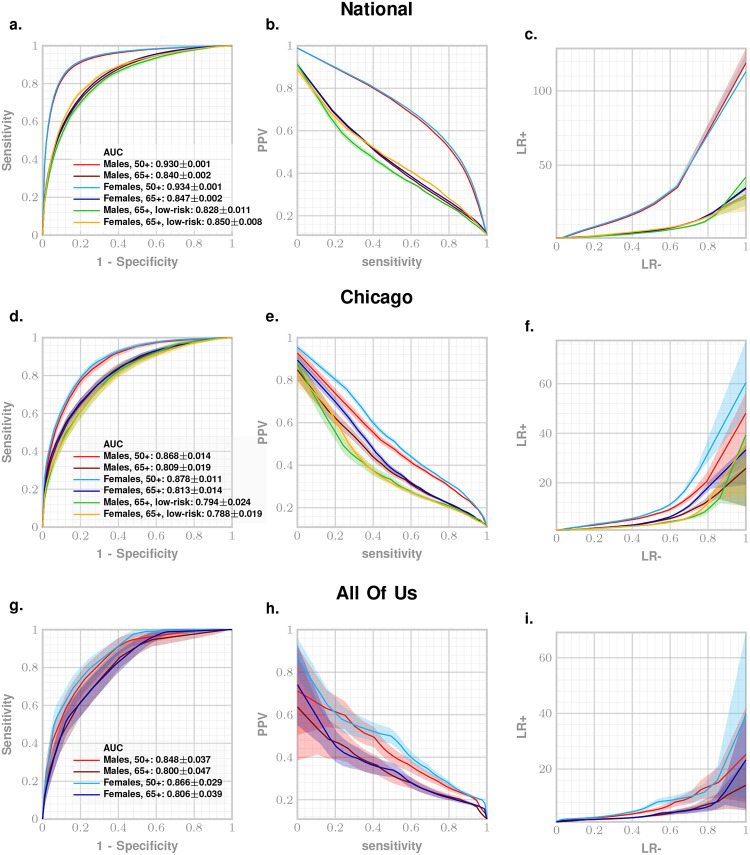
Predictive performance of ZeBRA. Panels demonstrate out-of-sample ROC curves, precision-recall curves, and likelihood ratio curves for National (a-c), Chicago (d-f), and All Of Us (g-i) datasets. Low-risk curves for the All Of Us dataset were not plotted due to the insufficent number of patients in the subcohorts.

Our main results are presented in Fig. 2 as ROC, precision-recall (PRC) and likelihood ratio (LR) curves for National, Chicago, and All Of Us datasets. Predictive performance for 50+ and 65+ age groups at 95% specificity setting is enumerated in Table II. We achieve out-of-sample AUC of 93% and 84% for both males and females on held-back samples from the National dataset for 50+ years and 65+ year sub-cohorts respectively (Figure 2).

Performance of ZeBRA stays robust across the other independent datasets reaching AUC of 87–88% for 50+ years old and 81% for 65+ years old subcohorts of Chicago dataset (Figure 2d), and AUC of 85–86% for 50+ years old and 80% AUC for 65+ years old cohorts for All Of Us dataset (Fig. 2g).

Translated into screening tool performance in primary care settings with a 95% specificity risk threshold, for every 100 patients to be diagnosed with ADRD in 1 year with no currently present risk factors of ADRD, ZeBRA raises flag on 104 patients, out of which 65 are true positives, 40 are false alarms, and 35 cases are missed.

Additionally, ZeBRA achieves AUC of 82–85% AUC for 65+ years old low risk subcohorts of National dataset and 79% AUC for 65+ years old low risk subcohorts of Chicago dataset. Translated into screening tool performance in primary care settings with a 95% specificity risk threshold, for every 100 patients to be diagnosed with ADRD in 1 year with no currently present risk factors of ADRD, ZeBRA raises flag on 72 patients, out of which 37 are true positives, 35 are false alarms, and 63 low-risk cases are missed.

**Figure 3.**
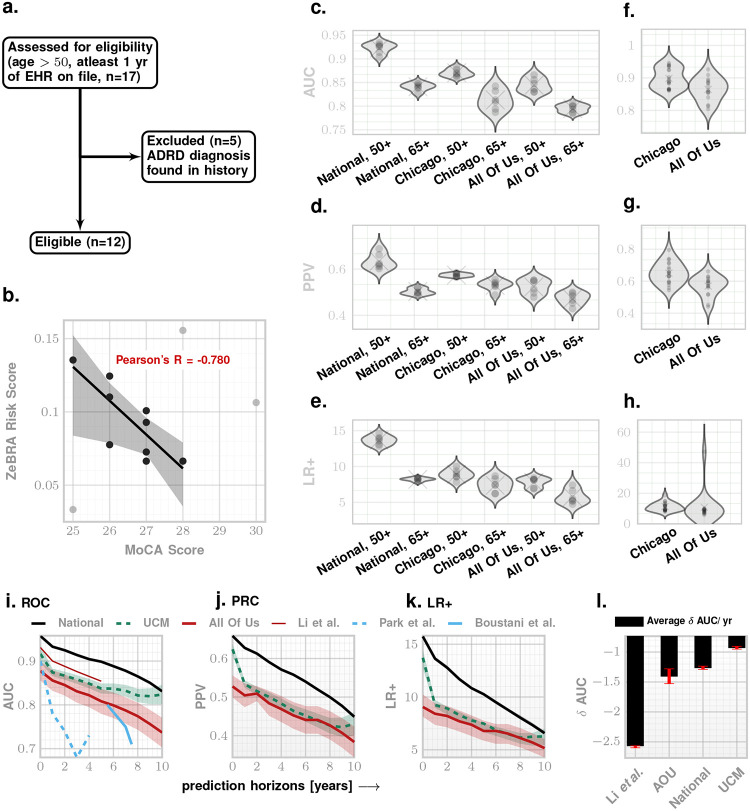
Prospective validation, demographic performance, and temporal generalization of ZeBRA. **a**, CONSORT diagram for the UCM Pilot cohort illustrating inclusion and exclusion criteria. **b**, Relationship between Montreal Cognitive Assessment (MoCA) scores and ZeBRA risk scores in the UCM Pilot study; lower MoCA values, indicating greater cognitive impairment, are strongly correlated with higher estimated risk (R=-0.78). **c–e**, Model performance by age subgroup (50+, 65+) across datasets, corresponding to Table II. **f–h**, Performance across demographic strata reported in Table III. **i–k**, Prediction performance at varying time horizons (1–10 years) compared with prior frameworks from the literature (Li *et al*., Park *et al*., and Boustani *et al*.), showing that ZeBRA maintains higher discrimination (ROC), precision–recall, and likelihood ratios over extended horizons and across independent cohorts (National, Chicago, and All Of Us). **l**, Average annual degradation in predictive discrimination (*δ*AUC/year) across datasets; ZeBRA exhibits a slower decay of approximately 1.0 − 1.3% per year compared with 2.6% for Li *et al*., sustaining AUC ≥ 0.83 at ten years.

Additionally, for the Chicago and All Of Us datasets (which include structured information on patients’ race, ethnicity, and sex) we report model performance across demographic subcohorts (Table III, Fig. 3c–h). Performance remains consistent across all reported demographic strata: sex (male, female), race (White, Black, Asian, Other), and ethnicity (Hispanic, non-Hispanic) groups (AUC 86% – 94% for the Chicago dataset, and 73% – 85% for the All Of Us dataset, Mean LR+ were 10.2 for Chicago and 8.9 for All Of Us). These findings are particularly relevant given persistent socioeconomic and demographic disparities in ADRD screening access and outcomes.

Our predictive performance expectedly degrades as we predict the onset of ADRD earlier. To assess this degradation of performance, we conducted a retrospective validation on National, Chicago, and All Of Us datasets with prediction horizon ranging from 0 to 10 years. For each horizon, we ensured that corresponding control cohort comprises patients who are not diagnosed with ADRD for a period that is at least one year longer than the prediction horizon (ranging from 1 years to 11 years respectively, Figure 3i–k). Our results show that ZeBRA AUC degrades approximately 1 – 1.3% per year. This degradation is slow enough to suggest that we can use ZeBRA with acceptable reliability to predict diagnoses up to 10 years into the future. Importantly, ZeBRA significantly outperforms reported ADRD studies that provide AUC for extended prediction horizons^15,43,44^ (Figure 3i–k and Table IV and Supplementary Table SI-V).

### Prospective Pilot:

Having established performance in retrospective data, we next evaluated ZeBRA ‘s clinical relevance via prospective correlation with cognitive scores (Fig. 3a–b). For our limited prospective validation, we collected EHR data as part of a pilot study conducted at the University of Chicago Medical Center to assess the risk of ADRD onset using ZeBRA and assessed participants in person using Montreal Cognitive Assessment (MoCA) score^45^. In this validation, we included all patients with available MoCA scores and corresponding EHR data, and excluded individuals with ADRD recorded in medical history (Fig. 3a).

MoCA assessment results were available in two formats: the full-scale version with a maximum score of 30 (MoCA-30), and a subset version excluding visual tasks, with a maximum score of 22 (MoCA-22). For consistency, all MoCA-22 scores were converted to the standard 30-point scale using a validated conversion method^46^.

We computed ZeBRA risk scores for these patients using the date of MoCA assessment as the index date, and an observation window of 2 years leading to the index date, and evaluated the association between ZeBRA-predicted risk of future ADRD and cognitive performance as measured by MoCA (Figure 3b): lower MoCA scores, indicating greater cognitive impairment were strongly correlated with elevated ZeBRA risk scores (Pearson’s R=-0.780).

### ZeBRA Comorbidity Patterns:

**Figure 4.**
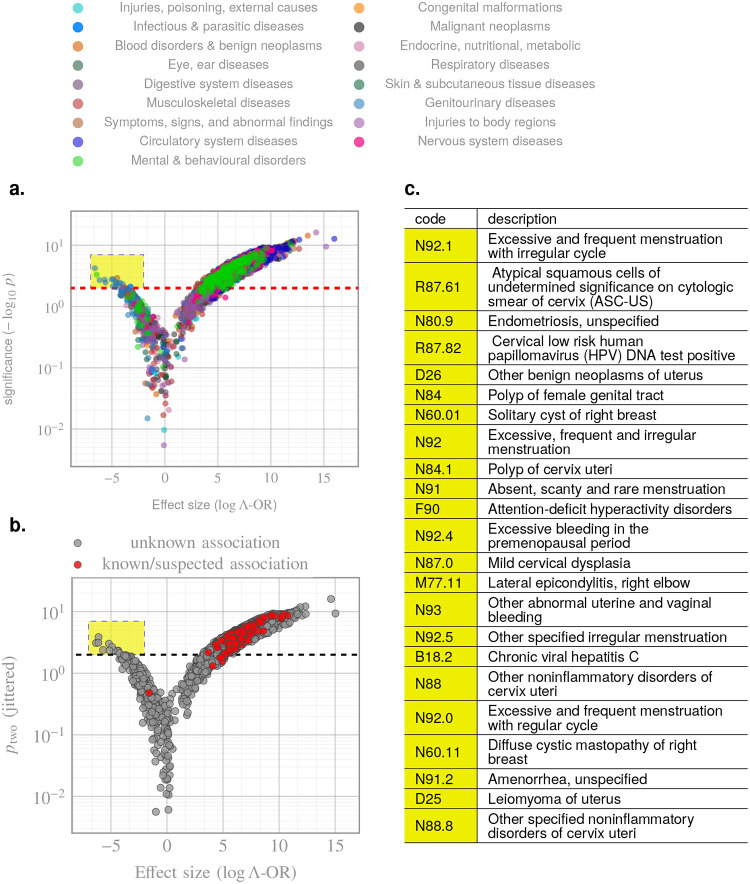
Volcano plot of Λ-OR for diagnostic codes modulating ZeBRA risk. **a**, Expected volcano pattern illustrating |Λ-OR| versus significance (-logp); canonical ADRD risk factors (e.g., cerebrovascular disease, hypertension, diabetes, obesity) rise to the upper right, as expected. **b**, Alignment of the highest-magnitude Λ-OR features with previously reported ADRD risk factors summarized in Table V, confirming consistency between model-derived effect sizes and epidemiologic literature. **c**, Highlighted points indicate apparent “protective” associations, largely corresponding to hormone-related gynecologic conditions discussed in the text.

Beyond predictive accuracy, we examined how individual ICD codes in patient histories modulate the risk of ADRD onset. To address potential label noise, arising from misdiagnosed cases and undiagnosed or late-diagnosed controls, we introduced a model-informed metric for effect-size estimation and feature attribution, termed the Λ-OR (see Online Methods). The resulting volcano plot (Fig. 4a) exhibited the expected pattern, with circulatory and nervous system diseases showing the most pronounced risk-increasing effects. We also identified a subset of codes with statistically significant protective associations (see Discussion). ZeBRA ‘s estimated impacts for known or suspected risk factors of ADRD are summarized in Table V, demonstrating strong alignment with previously reported comorbid associations. All codes whose prefixes match indications in Table V are highlighted in Fig. 4b, showing that such prior diagnoses are almost universally attributed as risk-increasing.

### Performance Comparison against existing models:

We benchmarked ZeBRA against prior EHR-based ADRD prediction frameworks from Li *et al*.^15^ and Wang *et al*.^35^ using the National dataset for direct comparison (Tables VI – VII). Li *et al*. evaluated four progressively stringent case definitions: **CP1** (one or more encounters with an ADRD diagnosis), **CP2** (two or more encounters), **CP3** (five or more encounters), and **CP4** (at least one ADRD diagnosis and one anti-dementia prescription). Across all definitions, ZeBRA demonstrated consistently higher discrimination. At the 1-year horizon, ZeBRA improved mean AUC by **5–8%** relative to Li *et al*. (e.g., 0.93 → 0.96 for CP1 and 0.90 → 0.95 for CP2), with performance advantages persisting through five years (**AUC** ≥ 0.91 for ZeBRA vs. 0.85 for Li). The difference widened further at longer horizons, where Li’s model degraded rapidly beyond three years (δ AUC/year ≈ −2.6%), while ZeBRA exhibited only gradual decay (δ AUC/year ≈ −1.0 to −1.3%), maintaining AUC ≥ 0.90 through five years and AUC ≥ 0.83 at ten years (Fig. 3l).

When compared with the large-language-model framework of Wang *et al*., which combined structured EHR input with in-context embedding refinement, ZeBRA achieved markedly higher precision and overall balance (Table VII). At the 1-year horizon, ZeBRA improved F1 scores from 0.31–0.34 to 0.56–0.60 on the same cohort, reflecting both superior discrimination and better calibration.

These comparisons, all performed on the National dataset, demonstrate that ZeBRA not only outperforms existing ADRD predictors in absolute accuracy and temporal stability but also retains calibrated, noise-corrected risk attributions via Λ-OR analysis, capabilities unavailable in conventional machine-learning or LLM-based approaches.

## DISCUSSION

We developed and validated ZeBRA, an automated, zero-burden screening tool for ADRD that leverages longitudinal comorbidity patterns in routine EHR data. Across sex, age, race/ethnicity, and baseline risk strata, ZeBRA accurately preempts incident ADRD for up to ten years before the first recorded diagnosis.

Across four independent datasets, ZeBRA achieved consistently high discrimination, with **AUCs of 0.93** ± **0.01 at 1 year** and **0.83** ± **0.01 at 10 years**, corresponding to **LR**^+^ > **10** at 95% specificity. Performance remained stable across **sex, age, race, and ethnicity** subgroups (Table III), indicating minimal demographic bias and strong generalizability. Notably, predictive strength persisted even within the **low-risk subcohort** lacking known ADRD comorbidities (AUC > 0.80), underscoring ZeBRA ‘s ability to detect latent preclinical signals invisible to conventional risk models. Importantly, temporal analysis revealed that ZeBRA ‘s discriminative power degrades only gradually over time, with a mean δAUC/year≈1.0–1.3% compared with ≈ 2.6% for Li *et al*., maintaining AUC ≥ 0.83 at ten years (Fig. 3l). This stability across horizons suggests that predictive signals encoded in comorbidity trajectories remain informative throughout the prolonged preclinical phase.

Using the lambda-Odds Ratio (Λ-OR) for noise-corrected attribution, we identified the comorbid indications with the strongest population-level associations (Fig. 4), including malignant neoplasms, congenital malformations, and diseases of the nervous, digestive, and integumentary systems. These signals align with previously reported ADRD risk factors and comorbidities^16–34^ (Table V), suggesting that ZeBRA offers dependable explainability: high-effect-size factors modulating ZeBRA risk align with established or suspected ADRD risk drivers in current clinical literature.

In addition, our Λ-OR analysis revealed several comorbidity codes with statistically significant “protective” associations (Fig. 4a). These predominantly comprise reproductive and gynecologic disorders including excessive or irregular menstruation, endometriosis, benign uterine and breast neoplasms, cervical polyps and dysplasia, and ovarian cysts. Although clinically heterogeneous, these conditions share a common endocrine substrate, namely prolonged or dysregulated exposure to endogenous estrogen. There is extensive epidemiologic and experimental evidence that cumulative lifetime estrogen signaling is neuroprotective^47^. Women with longer reproductive spans (later menopause, later age at last childbirth) exhibit reduced dementia risk, whereas abrupt loss of estrogen (e.g., surgical oophorectomy, premature menopause) is consistently associated with heightened risk^48–50^. Mechanistically, estrogen enhances synaptic plasticity, modulates hippocampal connectivity, reduces β-amyloid aggregation, improves mitochondrial function, and buffers oxidative stress—all pathways implicated in Alzheimer’s disease pathogenesis^51,52^. Thus, the appearance of these gynecologic codes as protective within our framework is biologically consistent with established estrogen-mediated resilience against neurodegeneration.

We observed ADHD as a protective comorbidity, which requires a more nuanced interpretation. ADHD is characterized by lifelong executive and attentional dysfunction involving frontostriatal circuits, domains that overlap substantially with those affected in MCI and early dementia^53,54^. In older adults, this overlap complicates differential diagnosis and has been described as a phenotypic mimic of prodromal neurodegeneration, raising the possibility of diagnostic substitution or delayed dementia coding in real-world clinical data. Thus, the negative association is unlikely to reflect true neurobiological protection and may instead capture care-pathway and ascertainment effects, whereby individuals with longstanding ADHD engage earlier with psychiatric or neurologic care and follow distinct diagnostic trajectories. A secondary, speculative explanation is differential exposure to psychostimulants, which increase dopaminergic and noradrenergic signaling/neuromodulatory systems supporting attention and executive function in aging^55^, although evidence that stimulant use modifies ADRD risk remains limited.

Importantly, ZeBRA does not posit causality; instead, it identifies latent comorbidity signatures that shift the likelihood surface for ADRD onset. The emergence of reproductive codes in the protective direction highlights the interpretability of the framework: clinically meaningful and biologically plausible signals surface naturally when the model is trained on unconstrained diagnostic histories.

### Positioning relative to prior EHR models:

The strongest previously reported EHR-based screeners include Li *et al*.^15^ (AUC = 90% at 1-year and 85% at 5-year windows) and Wang *et al*.^35^, who combined supervised learning with in-context LLM refinement (precision = .2442±.0247 at recall = .5560±.0524 at 1-year). Both represent important advances; however, neither reports performance across age bands, demographics, or high-/low-risk subcohorts, nor evaluation on datasets from independent health systems. These gaps are critical for clinical translation, given the known heterogeneity in EHR coding across sites. ZeBRA achieves the best overall performance among the three (Tables VI, VII), maintains robust accuracy up to ten years before diagnosis (versus five and three years, respectively), validates retrospectively on two independent datasets (Chicago, All Of Us) (Table II, Fig. 2), and demonstrates prospective concordance with cognitive screening (Fig. 3b). Notably, the average rate of AUC degradation with horizon (δAUC/year) was approximately 1.0% for ZeBRA compared with 2.6% for Li *et al*., confirming that predictive fidelity is preserved over longer time frames.

### Methodological contributions:

Although all three approaches are data-driven, ZeBRA addresses EHR sparsity, skew, and heterogeneity via (i) multi-channel embeddings (presence and odds ratio) for diagnoses, prescriptions, and procedures; (ii) an ensemble of LightGBM classifiers^38,39^; and (iii) hierarchical code groupings that enrich rather than truncate comorbidity representation. This design reduces vulnerability to coding inconsistency and incompleteness common in practice^56,57^. The study’s scale (N=12,971,707) exceeds that of Li (N=1,062,478) and Wang (N=29,557), representing, to our knowledge, the largest cohort used for EHR-based early screening of ADRD or any other condition. Finally, we introduce a model-informed, regularized effect-size estimator (Λ-OR) tailored to large observational datasets, complementing headline metrics with clinically interpretable signals.

### Clinical integration and use cases:

Without the requirement for laboratory testing, imaging, or questionnaires, ZeBRA can operate as a low-cost, near-instantaneous screen in primary care or specialty settings (e.g., neurology, geriatrics), either alone or adjunctive to brief, validated neuropsychological instruments as recommended by the American Academy of Neurology^58^, including within the Medicare Annual Wellness Visit cognitive assessment^59^. Such integration could help address underuse of cognitive screening in primary care, where time, workflow, and follow-up uncertainty remain barriers^13,60,61^. ZeBRA could also be applied to individuals with subjective memory concerns or incipient mild cognitive impairment who have not undergone biomarker evaluation, and for presymptomatic trial enrichment, where scalable, passive, and equitable identification of high-risk participants is a critical unmet need.

### Translational context:

Therapeutic progress in ADRD has been limited, and the amyloid cascade hypothesis remains debated^62–67^. With >130 disease-modifying trials active as of 2025 and modest effects from recent anti-amyloid agents^4,68^, shifting earlier—into the prolonged presymptomatic phase defined biologically by amyloid/tau positivity^7–10^—is a priority. Yet PET/CSF-based diagnostics are costly and operationally complex, and blood-based assays are not FDA-approved for routine use^4^. Passive, scalable screens are therefore essential^11–14,69–71^. By exploiting what already exists in the EHR, ZeBRA offers a pragmatic path to earlier identification, broader outreach, and more inclusive recruitment, potentially lowering costs and widening access to presymptomatic interventions.

### Limitations and future work:

This study is retrospective for National, Chicago, and All Of Us, and includes a limited prospective pilot; larger, multi-site prospective trials are needed to quantify impact on clinical decision-making, downstream testing, and health outcomes. Although we harmonize evaluation at a fixed prevalence (10.9%), site-specific calibration will be important for deployment. While we report performance across demographics and risk strata, formal fairness assessments (e.g., subgroup calibration, error parity) and health-economic analyses were beyond scope. Finally, Λ-OR offers population-level interpretability, but patient-level explanations and workflow-aligned thresholds (e.g., referral vs. watchful waiting) merit further study.

In conclusion, ZeBRA delivers accurate, generalizable, and interpretable ADRD risk screening across heterogeneous datasets—including two external retrospective cohorts and a prospective pilot—without requiring new tests. Its scalability, long-horizon stability, and low operational burden position it as a practical tool for population health management and for enriching presymptomatic clinical trials, complementing (rather than replacing) biomarker-based diagnostics and neuropsychological assessment.

## Online Methods

### Feature Engineering

We denote the observation window of the patient records contain of each patient x as:

(1) $$D=\{\text{DX},\text{RX},\text{PROC}\},\;\forall j\in D,\;W^{j}(x)=\left\{\left(c_{1}^{j},t_{1}^{j}\right),\ldots ,\left(c_{i}^{j},t_{i}^{j}\right),\ldots ,\left(c_{r}^{j},t_{r}^{j}\right)\right\},\;c_{i}^{j}\in C^{j}$$

where D is a set of used EHR data channels, $c_{i}^{j}$ are codes recorded in each channel, $t_{i}^{j}$ are time points for the recorded codes measuring weeks since the time of the first recorded DX code $t_{1}^{DX}$, and $C^{j}$ is the set of valid codes for each EHR data channel. We map each patient’s observation window to representations, namely the binary presence feature vector and the odds-ratio embedding vector, with each EHR data channel mapped separately, totaling 6 feature vectors per patient.

### Prescription Drug Coding

Prescription drug (RX) codes in our primary (National) dataset are provided in the National Drug Code (NDC) format^72^, which is designed to facilitate the identification and commercial distribution of pharmaceuticals. This coding system, as well as the widely adopted RxNorm^73^ emphasizes product-specific and manufacturer details of the generic drugs rather than their therapeutic classification and characteristics, thus hindering its use for EHR data analysis.

To facilitate the analysis of prescriptions according to their therapeutic effect, we developed a custom NDC-compatible coding scheme to convert all RX codes in the used datasets. This system, analogous to ICD-10 for diagnostic codes, progressively details therapeutic information of a generic drug with each successive character in the code. Each RX code begins with an “rx” prefix to enhance readability and facilitate parsing. This is followed by three alphanumeric characters encoding, respectively, the Therapeutic Group, Therapeutic Class, and Therapeutic Subclass, latter derived from the Therapeutic Class column in the RED BOOK. In cases where any of these attributes are missing or listed as NEC (i.e., not elsewhere classifiable), we use the placeholder character “X”. Finally, to identify a specific generic drug, we append a numeric identifier indicating its order within the set of drugs sharing the same initial five-letter therapeutic code (see Figure 1e for the example of generic drug coding).

Information on NDC codes and the corresponding therapeutic attributes of the generic drugs they represent is sourced from the Micromedex RED BOOK^©^^74^, which accompanies the Merative MarketScan dataset.

### Code Presence Embedding Vectors

For each EHR data channel j∈D, we define a binary presence embedding based on the patient observation window $W^{j}(x)$. Let $C^{j}$ denote the set of all distinct codes observed across the embedding inference set, augmented with hierarchical code prefixes to enable generalization across varying code granularities.

Specifically, we include for each code $c\in C^{j}$ its prefixes according to the following scheme:

- DX: prefixes of length 1, 2, 3, 5 and 6,
- RX: prefixes of length 3, 4, 5, and 8,
- PROC: prefixes of length 1, 2, 3, 4, and 5.

We define the augmented code set ${𝒞}^{j}$ for channel j as:

$${𝒞}^{j}=\{\text{all codes and specified-length prefixes observed in Code Inference Set}\}$$

Let ${𝒪}^{j}(x)$ be the set of codes and code prefixes present in the observation window $W^{j}(x)$ for patient x. Then, the presence vector $x_{j}^{\text{presence}}\in \{0,1{\}}^{\left|{𝒞}^{j}\right|}$ is defined elementwise as:

(2) $$\forall c_{i}\in {𝒞}^{j},\;x_{j,i}^{\text{presence}}(x)=\left\{\begin{array}{ll} 1, & \text{if}\;c_{i}\in {𝒪}^{j}(x)\\ 0, & \text{otherwise} \end{array}\right.$$

In total, the number of codes and code prefixes we track is 21,070 for DX channel, 2,156 for RX channel, and 17,261 for PROC channel. See Supplementary Table SI-VI for the total number of codes and prefix-based aggregations used in each presence model. The resulting binary vectors serve as input to the gradient boosting classifiers for each EHR channel.

### Odds Ratio Embedding Vectors

For each code $c\in C^{j}$, in each data channel j∈D, we compute the dictionary of cubed odds ratios based on patients from the Code Inference Set:

(3) $$\forall c\in C^{j},\;{\text{OR}}^{j}(c)={\left(\frac{P\left(\exists t\;\text{s.t.}\left(c,t\right)\in W^{j}\left(y\right)|y\in \text{cases}\right)}{P\left(\exists t\;\text{s.t.}(c,t)\in W^{j}(z)|z\in \text{controls}\right)}\right)}^{3}$$

Then we compute odds ratios for all codes in the patients’ observation windows:

(4) $$\forall j\in D,\;x_{j}^{\text{OR}}=\left\{{\text{OR}}^{j}(c),c\in W^{j}(x)\right\}$$

and finally map the computed odds ratios of each patient x to a 12-dimensional embedding via aggregation functions ${𝒜}_{k}$

(5) $$\forall j\in D,\;x_{j}^{\text{OR}\_\text{embedding}}=\left\{{𝒜}_{k}\left(x_{j}^{\text{OR}}\right)|k=1,\ldots ,12\right\}$$

See Supplementary Table SI-VII for the complete list of aggregation functions ${𝒜}_{k}$.

### Posterior Odds Ratios

Large-scale observational datasets — spanning genomics biobanks, administrative claims, and electronic health records (EHR) — increasingly drive scientific discovery and risk modeling. Odds ratios (ORs) and related effect-size estimates remain the dominant tools for summarizing exposure-outcome associations. Yet as cohort sizes reach hundreds of thousands or millions, naive OR analysis breaks down: effect sizes inflate, p-values vanish, and nearly all features appear statistically significant, even when no true association exists.

This pathology reflects two structural vulnerabilities of large observational studies:

1. **Label Noise:** Case/control labels are often derived from imperfect sources such as diagnostic codes or weak classifiers, introducing systematic misclassification^75^.
2. **Large Sample Size Effects:** As n→∞, variance estimates collapse, magnifying even trivial fluctuations or structural biases, yielding spurious discoveries^76,77^.

In randomized trials, ORs approximate causal contrasts under minimal assumptions. In contrast, observational settings lack experimental control; naive OR computations applied directly to raw exposure-outcome tables, without adjustment for label noise or structural confounding, produce exaggerated significance that worsens with scale.

Post-hoc attribution methods such as SHAP and LIME^78^ offer model-agnostic feature importance scores, but inherit related limitations: they remain artificially stable under weak, non-predictive models, overlook interaction-driven effects, and fail to correct for misclassification or instability^79^.

Existing corrections for OR bias primarily target small-sample misclassification adjustment^76^, or require external gold-standard validation cohorts, which are rarely available at scale.

We introduce the *lambda-Odds Ratio (*Λ-OR), a model-informed, asymptotically faithful method for effect-size estimation and feature attribution tailored to large, noisy observational datasets. Λ-OR differs from classical OR estimation:

1. Individuals are stratified by calibrated model-predicted risk, forming empirical high- and low-risk cohorts.
2. Exposure odds contrasts are computed across these strata, rather than from unstratified marginal tables.
3. A ridge-regularized inversion corrects for outcome misclassification and ensures numerical stability, even under near-degenerate class distributions.

We consider retrospective observational studies where binary case/control labels $\tilde{Y}\in \{0,1\}$ are derived from imperfect classifiers or noisy diagnostic records (e.g., EHR-derived phenotypes), and exposure status X∈{0,1} is assumed to be known without error. Let the observed (noisy) 2-by-2 contingency table be

(6) $$\tilde{T}=\left[\begin{array}{ll} \tilde{a} & \tilde{b}\\ \tilde{c} & \tilde{d} \end{array}\right],$$

where $\tilde{a}$ denotes the count of individuals with X=1 and $\tilde{Y}=1$, and so on. These observed labels are subject to misclassification governed by sensitivity $p=P(\tilde{Y}=1|Y=1)$ and specificity $q=P(\tilde{Y}=0|Y=0)$, where Y denotes the true latent label.

The misclassification process is represented by the stochastic matrix

(7) $$K=\left[\begin{array}{cc} p & 1-p\\ 1-q & q \end{array}\right],$$

so that the observed counts satisfy, in expectation,

(8) $$\tilde{T}=TK^{⊤},$$

where T is the true (unknown) count matrix under perfect case/control assignment.

#### Ill-posed Inversion and the Need for Regularization:

Inversion of $K^{⊤}$ to estimate T via $T=\tilde{T}{\left(K^{⊤}\right)}^{-1}$ can result in infeasible solutions where the recovered cell counts are negative or numerically unstable, particularly when K is nearly singular or p+q≈1. To address this, we adopt a ridge-regularized inverse:

(9) $$T^{\left(\lambda \right)}≜\tilde{T}{\left(K^{⊤}+\lambda I\right)}^{-1},$$

where λ>0 is chosen to ensure all entries of $T^{\left(\lambda \right)}$ remain nonnegative and greater than a small threshold ϵ, preserving interpretability as approximate counts. The minimal such λ, if it exists, defines our feasible correction. The regularized corrected contingency table is denoted

(10) $$T^{\left(\lambda \right)}=\left[\begin{array}{ll} a & b\\ c & d \end{array}\right],$$

and the corrected log-odds ratio is

(11) $$\text{log}\;\Lambda {\text{-OR}}^{\left(\lambda \right)}=\text{log}\left(\frac{ad}{bc}\right).$$

We estimate the standard error using the delta method, optionally incorporating the uncertainty in p, q (from validation data) via an analytic extra-variance term.

This framework generalizes classical odds ratio inference by explicitly modeling outcome misclassification and providing a consistent correction even when label quality varies or depends on classifier thresholds.

#### Interpretation:

This approach defines a class of corrected log-odds ratio estimators indexed by λ, each balancing fidelity to the noisy data against feasibility under misclassification uncertainty. In the limit λ→0, the estimator recovers the unregularized inverse, which may become undefined or yield negative entries. The practical estimator uses the smallest λ such that all recovered counts exceed a small threshold ϵ>0, typically ϵ∈[0.1,1].

This formulation enables consistent odds ratio estimation in large-scale electronic health record (EHR) studies, where label noise is prevalent and direct validation of every case/control label is infeasible.

#### Λ-OR *Variance Estimation:*

Under the assumption of independent cell counts, the classical variance of the log-odds ratio is

(12) $${\text{Var}}_{\text{naive}}\left(\text{log}\;\Lambda \text{-OR}\right)=\frac{1}{a}+\frac{1}{b}+\frac{1}{c}+\frac{1}{d}.$$

However, when the table $T^{\left(\lambda \right)}$ is obtained via inversion from noisy labels, additional uncertainty arises from the estimation of the misclassification rates p and q. Let $n_{\text{val}}$ denote the size of the validation cohort used to estimate p and q, assumed independent of the main study population. Define the extra-variance component via the delta method as

(13) $${\text{Var}}_{\text{extra}}=\left({\left(\frac{1-q}{(p+q-1{)}^{2}}\right)}^{2}\frac{p(1-p)}{n_{\text{val}}}\right.\left.+{\left(\frac{1-p}{(p+q-1{)}^{2}}\right)}^{2}\frac{q(1-q)}{n_{\text{val}}}\right)$$

where $n_{\text{val}}>1$. The total variance is given by

(14) $${\text{Var}}_{\text{total}}\left(\text{log}\;\Lambda \text{-OR}\right)={\text{Var}}_{\text{naive}}+{\text{Var}}_{\text{extra}}.$$

The corrected Wald statistic is: $Z=\frac{\text{log}\;\Lambda {\text{-OR}}^{\left(\lambda \right)}}{\sqrt{{\text{Var}}_{\text{total}}}}$,, with a two-sided p-value obtained from the standard normal tail: $p_{\text{two}}=2\Phi (-|Z|)$, with the (1-α) confidence interval:

$$\left[\text{log}\;\Lambda {\text{-OR}}^{\left(\lambda \right)}-z_{\alpha /2}\;\sqrt{{\text{Var}}_{\text{total}}},\text{log}\;\Lambda {\text{-OR}}^{\left(\lambda \right)}+z_{\alpha /2}\;\sqrt{{\text{Var}}_{\text{total}}}\right]$$

using $z_{\alpha /2}$ as the critical value from the standard normal distribution.

## Supplementary Files

This is a list of supplementary files associated with this preprint. Click to download.

- SI.pdf

## Figures and Tables

**Table I:** Patient Characteristics for all cohorts

Feature	National (n = 12,869,480)	Chicago (n = 72,650)	All Of Us (n = 29,565)	UCM Pilot (n = 12)
Target	Case: 484,721 (3.8%), Control: 12,384,759 (96.2%)	Case: 2,914 (4.0%), Control: 69,736 (96.0%)	Case: 354 (1.2%), Control: 29,211 (98.8%)	N/A
Sex	Male: 5,772,090 (44.9%), Female: 7,097,390 (55.1%)	Male: 30,731 (42.3%), Female: 41,919 (57.7%)	Male: 12,671 (42.9%), Female: 16,894 (57.1%)	Male: 3 (25.0%), Female: 9 (75.0%)
Race	N/A	Black/African-American: 34,225 (47.1%), White: 33,942 (46.7%), More than one Race: 811 (1.1%), Native Hawaiian/Pacific Islander: 65 (0.1%), Asian/Mideast Indian: 1,434 (2.0%), Native American/Alaska Native: 173 (0.2%), Unknown: 2,000 (2.8%)	White: 22,129 (74.8%), African American: 3,480 (11.8%), Asian/Mideast Indian: 386 (1.3%), More than one Race: 742 (2.5%), Unknown: 2,828 (9.6%)	Black/African-American: 7 (58.3%), White: 5 (41.7%)
Ethnicity	N/A	Hispanic or Latino: 2,558 (3.5%), Not Hispanic or Latino: 67,613 (93.1%), Unknown: 2,479 (3.4%)	Hispanic or Latino: 1,815 (6.1%), Not Hispanic or Latino: 26,755 (90.5%), Unknown: 995 (3.4%)	Hispanic or Latino: 1 (8.3%), Not Hispanic or Latino: 10 (83.3%), Unknown: 1 (8.3%)
Mean age at screening	61 years 7 months	65 years 1 month	64 years 4 months	87 years 4 months
Type 2 Diabetes Mellitus (E11)	Case: 177,240 (36.6%), Control: 2,543,094 (20.5%)	Case: 442 (15.2%), Control: 3,665 (5.3%)	Case: 177 (50.0%), Control: 6,793 (23.3%)	3 (25.0%)
Overweight and Obesity (E66)	Case: 63,820 (13.2%), Control: 1,484,910 (12.0%)	Case: 154 (5.3%), Control: 2,537 (3.6%)	Case: 153 (43.2%), Control: 6,407 (21.9%)	1 (8.3%)
Depressive Episode (F32)	Case: 85,970 (17.7%), Control: 976,730 (7.9%)	Case: 164 (5.6%), Control: 1,050 (1.5%)	Case: 162 (45.8%), Control: 5,432 (18.6%)	0 (0.0%)
Essential (Primary) Hypertension (I10)	Case: 395,336 (81.6%), Control: 6,531,073 (52.7%)	Case: 828 (28.4%), Control: 8,333 (11.9%)	Case: 301 (85.0%), Control: 15,290 (52.3%)	6 (50.0%)
Chronic Ischemic Heart Disease (I25)	Case: 184,485 (38.1%), Control: 1,495,981 (12.1%)	Case: 283 (9.7%), Control: 2,189 (3.1%)	Case: 151 (42.7%), Control: 4,477 (15.3%)	0 (0.0%)
Heart Failure (I50)	Case: 97,591 (20.1%), Control: 437,233 (3.5%)	Case: 268 (9.2%), Control: 1,473 (2.1%)	Case: 85 (24.0%), Control: 1,360 (4.7%)	0 (0.0%)
Cerebral Infarction (I63)	Case: 56,193 (11.6%), Control: 187,148 (1.5%)	Case: 121 (4.2%), Control: 372 (0.5%)	Case: 48 (13.6%), Control: 969 (3.3%)	0 (0.0%)
Chronic Obstructive Pulmonary Diseases (J41-J44)	Case: 115,541 (23.8%), Control: 859,383 (6.9%)	Case: 210 (7.2%), Control: 1,442 (2.1%)	Case: 88 (24.9%), Control: 2,145 (7.3%)	0 (0.0%)
Interstitial Pulmonary Diseases (J84.1, J84.89, J84.9)	Case: 20,266 (4.2%), Control: 124,749 (1.0%)	Case: 17 (0.6%), Control: 346 (0.5%)	Case: 19 (5.4%), Control: 416 (1.4%)	0 (0.0%)
Osteoporosis Without Current Pathological Fracture (M80-M81)	Case: 103,045 (21.3%), Control: 940,260 (7.6%)	Case: 132 (4.5%), Control: 1,055 (1.5%)	Case: 112 (31.6%), Control: 3,693 (12.6%)	3 (25.0%)

**Table II:** ZeBRA performance (at 95% specificity)

Dataset	Age	Sex	Cohort	Sensitivity	PPV	NPV	AUC	LR+	LR−
National	50+	Male	all	0.68 ± 0.00	0.62 ± 0.00	0.96 ± 0.00	0.930 ± 0.001	13.59 ± 0.07	0.34 ± 0.00
			high-risk	0.65 ± 0.00	0.61 ± 0.00	0.96 ± 0.00	0.926 ± 0.002	13.07 ± 0.08	0.37 ± 0.00
			low-risk	0.70 ± 0.02	0.66 ± 0.01	0.96 ± 0.00	0.904 ± 0.008	13.86 ± 0.29	0.31 ± 0.02
		Female	all	0.69 ± 0.00	0.62 ± 0.00	0.96 ± 0.00	0.934 ± 0.001	14.01 ± 0.06	0.33 ± 0.00
			high-risk	0.66 ± 0.00	0.60 ± 0.00	0.96 ± 0.00	0.927 ± 0.001	12.86 ± 0.06	0.36 ± 0.00
			low-risk	0.75 ± 0.01	0.69 ± 0.00	0.97 ± 0.00	0.914 ± 0.006	14.62 ± 0.22	0.27 ± 0.01
	65+	Male	all	0.40 ± 0.01	0.49 ± 0.00	0.93 ± 0.00	0.840 ± 0.002	8.50 ± 0.09	0.63 ± 0.00
			high-risk	0.41 ± 0.00	0.49 ± 0.00	0.93 ± 0.00	0.838 ± 0.003	8.16 ± 0.09	0.62 ± 0.01
			low-risk	0.37 ± 0.02	0.52 ± 0.01	0.93 ± 0.00	0.827 ± 0.011	7.81 ± 0.39	0.66 ± 0.02
		Female	all	0.42 ± 0.00	0.50 ± 0.00	0.93 ± 0.00	0.847 ± 0.002	8.22 ± 0.07	0.61 ± 0.00
			high-risk	0.41 ± 0.00	0.50 ± 0.00	0.93 ± 0.00	0.843 ± 0.002	8.19 ± 0.07	0.62 ± 0.00
			low-risk	0.42 ± 0.02	0.52 ± 0.01	0.93 ± 0.00	0.850 ± 0.008	8.36 ± 0.29	0.61 ± 0.02
Chicago	50+	Male	all	0.45 ± 0.03	0.57 ± 0.02	0.93 ± 0.00	0.868 ± 0.015	8.56 ± 0.56	0.58 ± 0.03
			high-risk	0.50 ± 0.05	0.58 ± 0.03	0.94 ± 0.01	0.870 ± 0.025	9.02 ± 0.94	0.53 ± 0.06
			low-risk	0.43 ± 0.04	0.57 ± 0.02	0.93 ± 0.00	0.861 ± 0.018	7.84 ± 0.66	0.60 ± 0.04
		Female	all	0.48 ± 0.02	0.58 ± 0.01	0.94 ± 0.00	0.877 ± 0.011	9.37 ± 0.45	0.55 ± 0.02
			high-risk	0.51 ± 0.04	0.58 ± 0.02	0.94 ± 0.00	0.883 ± 0.017	10.01 ± 0.76	0.52 ± 0.04
			low-risk	0.40 ± 0.03	0.57 ± 0.02	0.93 ± 0.00	0.864 ± 0.014	8.60 ± 0.60	0.63 ± 0.03
	65+	Male	all	0.36 ± 0.03	0.54 ± 0.02	0.92 ± 0.00	0.809 ± 0.019	7.38 ± 0.62	0.67 ± 0.03
			high-risk	0.33 ± 0.05	0.54 ± 0.04	0.92 ± 0.01	0.832 ± 0.032	8.68 ± 1.37	0.69 ± 0.05
			low-risk	0.32 ± 0.04	0.52 ± 0.03	0.92 ± 0.00	0.794 ± 0.023	6.23 ± 0.71	0.72 ± 0.04
		Female	all	0.38 ± 0.02	0.56 ± 0.02	0.93 ± 0.00	0.813 ± 0.014	8.28 ± 0.51	0.65 ± 0.03
			high-risk	0.46 ± 0.04	0.53 ± 0.02	0.94 ± 0.00	0.842 ± 0.021	7.49 ± 0.64	0.57 ± 0.04
			low-risk	0.33 ± 0.03	0.49 ± 0.02	0.92 ± 0.00	0.787 ± 0.019	6.21 ± 0.53	0.70 ± 0.03
All Of Us	50+	Male	all	0.40 ± 0.08	0.51 ± 0.05	0.93 ± 0.01	0.848 ± 0.035	8.11 ± 1.52	0.63 ± 0.08
			high-risk	0.38 ± 0.08	0.48 ± 0.05	0.93 ± 0.01	0.830 ± 0.039	6.98 ± 1.39	0.65 ± 0.08
		Female	all	0.48 ± 0.07	0.55 ± 0.04	0.94 ± 0.01	0.866 ± 0.028	8.39 ± 1.23	0.55 ± 0.08
			high-risk	0.31 ± 0.07	0.55 ± 0.05	0.92 ± 0.01	0.836 ± 0.032	8.19 ± 1.75	0.72 ± 0.07
			low-risk	0.50 ± 0.40	0.78 ± 0.23	0.94 ± 0.04	0.877 ± 0.114	26.51 ± 21.21	0.51 ± 0.41
	65+	Male	all	0.25 ± 0.07	0.48 ± 0.07	0.91 ± 0.01	0.800 ± 0.044	7.41 ± 2.08	0.78 ± 0.07
			high-risk	0.25 ± 0.07	0.47 ± 0.07	0.91 ± 0.01	0.793 ± 0.045	6.63 ± 1.85	0.78 ± 0.07
		Female	all	0.31 ± 0.07	0.50 ± 0.05	0.92 ± 0.01	0.806 ± 0.038	5.50 ± 1.21	0.73 ± 0.07
			high-risk	0.32 ± 0.07	0.50 ± 0.05	0.92 ± 0.01	0.785 ± 0.039	4.75 ± 1.03	0.73 ± 0.08
			low-risk	0.50 ± 0.40	0.63 ± 0.24	0.94 ± 0.05	0.77 ± 0.200	9.45 ± 7.56	0.53 ± 0.42

**Table III:** Consistent ZeBRA performance by demographics at 95% specificity.

Dataset	Race	Ethnicity	Sex	Sensitivity	PPV	NPV	AUC	LR+	LR−
Chicago	White	Not Hispanic Latino	TOTAL	0.45 ± 0.04	0.59 ± 0.02	0.93 ± 0.00	0.861 ± 0.021	8.79 ± 0.81	0.58 ± 0.04
			Male	0.39 ± 0.06	0.63 ± 0.04	0.93 ± 0.01	0.862 ± 0.028	8.82 ± 1.29	0.64 ± 0.06
			Female	0.50 ± 0.06	0.63 ± 0.03	0.94 ± 0.01	0.865 ± 0.030	9.53 ± 1.13	0.53 ± 0.06
		Hispanic Latino	TOTAL	0.60 ± 0.14	0.65 ± 0.06	0.95 ± 0.02	0.888 ± 0.057	12.12 ± 2.77	0.42 ± 0.15
			Male	0.60 ± 0.22	0.66 ± 0.09	0.95 ± 0.03	0.902 ± 0.068	12.02 ± 4.30	0.42 ± 0.23
			Female	0.64 ± 0.18	0.69 ± 0.07	0.96 ± 0.02	0.889 ± 0.074	11.71 ± 3.23	0.38 ± 0.19
	African American	Not Hispanic Latino	TOTAL	0.43 ± 0.02	0.56 ± 0.01	0.93 ± 0.00	0.865 ± 0.010	8.37 ± 0.41	0.60 ± 0.02
			Male	0.40 ± 0.04	0.54 ± 0.02	0.93 ± 0.00	0.864 ± 0.018	8.92 ± 0.79	0.63 ± 0.04
			Female	0.41 ± 0.03	0.59 ± 0.02	0.93 ± 0.00	0.867 ± 0.012	9.17 ± 0.56	0.62 ± 0.03
		Unknown	TOTAL	0.42 ± 0.28	0.68 ± 0.16	0.93 ± 0.03	0.937 ± 0.040	13.20 ± 8.84	0.60 ± 0.29
			Female	0.46 ± 0.29	0.66 ± 0.15	0.94 ± 0.03	0.938 ± 0.041	11.75 ± 7.61	0.57 ± 0.31
	Other	Not Hispanic Latino	TOTAL	0.53 ± 0.12	0.58 ± 0.06	0.94 ± 0.01	0.883 ± 0.050	9.06 ± 2.06	0.50 ± 0.13
			Male	0.56 ± 0.19	0.63 ± 0.09	0.95 ± 0.02	0.896 ± 0.070	11.23 ± 3.79	0.47 ± 0.20
			Female	0.54 ± 0.16	0.60 ± 0.07	0.94 ± 0.02	0.884 ± 0.055	7.97 ± 2.32	0.50 ± 0.17
		Hispanic Latino	TOTAL	0.56 ± 0.19	0.71 ± 0.08	0.95 ± 0.02	0.924 ± 0.042	13.64 ± 4.60	0.46 ± 0.20
			Male	0.63 ± 0.24	0.72 ± 0.10	0.95 ± 0.03	0.934 ± 0.048	9.68 ± 3.67	0.40 ± 0.25
			Female	0.64 ± 0.29	0.74 ± 0.11	0.96 ± 0.03	0.925 ± 0.059	14.77 ± 6.60	0.38 ± 0.30
		Unknown	TOTAL	0.39 ± 0.27	0.80 ± 0.16	0.93 ± 0.03	0.946 ± 0.022	18.82 ± 12.94	0.63 ± 0.27
			Female	0.40 ± 0.30	0.74 ± 0.18	0.93 ± 0.03	0.941 ± 0.036	14.80 ± 11.24	0.62 ± 0.31
All Of Us	White	Not Hispanic Latino	TOTAL	0.43 ± 0.07	0.59 ± 0.04	0.93 ± 0.01	0.863 ± 0.029	8.87 ± 1.36	0.60 ± 0.07
			Male	0.42 ± 0.09	0.57 ± 0.06	0.93 ± 0.01	0.857 ± 0.042	8.46 ± 1.88	0.61 ± 0.10
			Female	0.47 ± 0.09	0.63 ± 0.05	0.94 ± 0.01	0.876 ± 0.036	9.06 ± 1.82	0.56 ± 0.10
	African American	Not Hispanic Latino	TOTAL	0.42 ± 0.13	0.63 ± 0.08	0.93 ± 0.01	0.853 ± 0.055	9.39 ± 2.79	0.60 ± 0.13
			Male	0.32 ± 0.21	0.52 ± 0.19	0.92 ± 0.02	0.804 ± 0.113	9.82 ± 6.50	0.71 ± 0.22
			Female	0.48 ± 0.16	0.61 ± 0.08	0.94 ± 0.02	0.884 ± 0.045	9.64 ± 3.14	0.55 ± 0.16
	Other	NotHispanic Latino	TOTAL	0.47 ± 0.25	0.60 ± 0.15	0.94 ± 0.03	0.886 ± 0.065	7.53 ± 4.07	0.57 ± 0.27
			Male	0.25 ± 0.28	0.66 ± 3.21	0.92 ± 0.03	0.909 ± 0.074	47.25 ± 51.98	0.75 ± 0.30
			Female	0.57 ± 0.37	0.60 ± 0.18	0.95 ± 0.04	0.891 ± 0.096	8.11 ± 5.20	0.46 ± 0.39
		Hispanic Latino	TOTAL	0.49 ± 0.15	0.59 ± 0.08	0.94 ± 0.02	0.816 ± 0.068	6.56 ± 1.96	0.55 ± 0.16
			Male	0.43 ± 0.26	0.57 ± 0.16	0.93 ± 0.03	0.813 ± 0.124	5.77 ± 3.49	0.62 ± 0.28
			Female	0.52 ± 0.18	0.44 ± 0.09	0.94 ± 0.02	0.840 ± 0.064	7.01 ± 2.39	0.52 ± 0.19
		Unknown	TOTAL	0.35 ± 0.23	0.52 ± 0.13	0.92 ± 0.03	0.895 ± 0.061	8.22 ± 5.29	0.68 ± 0.24
			Male	0.55 ± 0.29	0.56 ± 0.14	0.94 ± 0.03	0.926 ± 0.035	10.05 ± 5.42	0.48 ± 0.31
			Female	0.50 ± 0.40	0.45 ± 0.21	0.94 ± 0.05	0.856 ± 0.119	7.05 ± 5.64	0.54 ± 0.43

**Table IV:** Comparision of performance across prediction horizon.

Prediction Window (years)	ZeBRA (National)	ZeBRA (Chicago)	ZeBRA (All Of Us)	Li et al.	Park et al.	Boustani et al.
0	0.957 ± 0.001	0.916 ± 0.020	0.877 ± 0.022	0.911 ± 0.001	0.900	
1	0.933 ± 0.001	0.874 ± 0.009	0.856 ± 0.024	0.876 ± 0.001	0.780	
2	0.925 ± 0.001	0.867 ± 0.009	0.845 ± 0.024		0.740	
3	0.914 ± 0.001	0.858 ± 0.010	0.831 ± 0.026	0.854 ± 0.002	0.680	
4	0.904 ± 0.001	0.849 ± 0.011	0.822 ± 0.027		0.730	
5	0.899 ± 0.002	0.837 ± 0.012	0.808 ± 0.028	0.831 ± 0.004		
5.5						0.800
6	0.888 ± 0.002	0.837 ± 0.012	0.799 ± 0.029			
7	0.878 ± 0.002	0.832 ± 0.013	0.787 ± 0.030			0.750
7.5						0.710
8	0.865 ± 0.002	0.821 ± 0.015	0.774 ± 0.031			
9	0.850 ± 0.003	0.820 ± 0.018	0.756 ± 0.032			
10	0.83 ± 0.003	0.824 ± 0.022	0.737 ± 0.034			

**Table V:** Corrected ZeBRA-obtained odds ratios for known ADRD risk factors.

Condition	ICD10 Codes	Mean(Log(SPOR)) / Std(Log(SPOR))	Source
Hyperparathyroidism	E21	14.118	Mathur et al., 2022^16^
Sepsis	A40-A41	10.535	Zeng et al., 2024^17^
Hypertensitve Heart and Chronic Kidney Disease	I12, I13	9.718	Kelly and Rothwell, 2022^18^
Traumatic Brain Injury	S06	8.841	Gu et al., 2022^19^
Hypo-osmolality and hyponatremia	E87.1	7.213	Chung et al., 2017^20^
Hypertension	I10, I11	6.180	Carey and Fossati, 2023^21^
Hyperlipidemia	E78	6.000	Ezkurdia et al., 2023^22^
Hypoparathyroidism	E20	5.615	Sikjaer et al., 2024^23^
Atrial Fibrillation	I48	5.511	Papanastasiou et al., 2021^24^
Major Depressive Disorder	F32, F33	5.432	Sáiz-Vázquez et al., 2021^25^
Cerebrovascular Disease	I60-I69	5.395	Love and Miners, 2016^26^
Hearing Loss	H90, H91	4.693	Litke et al., 2021^27^
Atherosclerosis	I25	4.620	Nordestgaard et al., 2022^28^
Type 2 Diabetes Mellitus	E11	4.434	Athanasaki et al., 2022^29^
Chronic Obstructive Pulmonary Diseases	J41-J44	4.427	Wang et al., 2022^30^
Age-related cataract	H25	3.930	Jun et al., 2012^31^
Obesity	E66	2.791	Beydoun et al., 2008^32^
Sleep Disorders	G47	2.277	Choe et al., 2022^33^
Alcohol Dependence/Abuse	F10	Not Observed	Xu et al., 2017^34^

**Table VI:** Performance comparison with Li et al. (95% CI)^15^

Target	Model	0-year AUC	1-year AUC	3-year AUC	5-year AUC
CP1 (1+ Encounter with ADRD Dx)	Li et al.	0.93 (0.93, 0.93)	0.90 (0.90, 0.90)	0.87 (0.87, 0.87)	0.85 (0.85, 0.85)
	ZeBRA	0.96 (0.96, 0.96)	0.93 (0.93, 0.94)	0.92 (0.91, 0.92)	0.91 (0.90, 0.91)
CP2 (2+ Encounters with ADRD Dx)	Li et al.	0.93 (0.93, 0.93)	0.90 (0.90, 0.90)	0.88 (0.88, 0.88)	0.86 (0.86, 0.86)
	ZeBRA	0.97 (0.97, 0.97)	0.95 (0.94, 0.95)	0.93 (0.92, 0.93)	0.91 (0.91, 0.92)
CP3 (5+ Encounters with ADRD Dx)	Li et al.	0.94 (0.94, 0.94)	0.90 (0.90, 0.90)	0.88 (0.88, 0.88)	0.85 (0.85, 0.85)
	ZeBRA	0.97 (0.97, 0.97)	0.95 (0.95, 0.95)	0.93 (0.93, 0.94)	0.92 (0.91, 0.92)
CP4 (1+ Encounter with ADRD Dx AND 1+ Anti-Dementia Rx)	Li et al.	0.91 (0.91, 0.91)	0.87 (0.87, 0.87)	0.85 (0.85, 0.85)	0.83 (0.83, 0.83)
	ZeBRA	0.97 (0.97, 0.97)	0.94 (0.93, 0.94)	0.91 (0.91, 0.91)	0.89 (0.89, 0.90)

**Table VII:** Performance Comparison with Wang *et al*.^35^

Prediction Window	Target	Model	Precision	Recall	F1 Score
0 Years	CP1	Wang et al.	.2494 ± .0268	.4860 ± .0427	.3271 ± .0150
		ZeBRA	.6841 ± .1968	.4860	.5652 ± .0594
	CP2	Wang et al.	.2609 ± .0162	.4980 ± .0801	.3407 ± .0257
		ZeBRA	.6831 ± .1893	.4980	.5777 ± .0603
1 Year	CP1	Wang et al.	.2157 ± .0231	.5300 ± .0379	.3064 ± .0285
		ZeBRA	.6414 ± .0906	.5300	.5823 ± .0363
	CP2	Wang et al.	.2442 ± .0247	.5560 ± .0524	.3386 ± .0294
		ZeBRA	.6387 ± .0785	.5560	.5952 ± .0336
3 Years	CP1	Wang et al.	.2248 ± .0133	.5220 ± .0601	.3136 ± .0199
		ZeBRA	.5941 ± .0346	.5220	.5563 ± .0165
	CP2	Wang et al.	.2375 ± .0140	.5140 ± .0174	.3247 ± .0144
		ZeBRA	.5948 ± .0381	.5140	.5540 ± .0178

## Data Availability

The individual-level datasets used for model training and validation are not publicly available and are owned and governed by their respective data custodians (including national administrative claims and institutional health system partners); access is subject to data-use agreements and regulatory approvals and therefore cannot be shared by the authors. To support transparency and reproducibility, we will provide a working implementation of the ZeBRA model via a publicly accessible API deployed on Google Cloud. The API endpoint, together with detailed usage instructions, an example JSON input schema, and scripts demonstrating how to generate predictions and reproduce reported summary-level analyses, is available in a dedicated GitHub repository at https://github.com/zeroknowledgediscovery/zebra_adrd, and on zenodo with a persistent DOI (https://doi.org/10.5281/zenodo.18486649). This repository will enable independent evaluation of model behavior on external datasets while respecting data ownership, privacy, and contractual constraints.
